# BCL-XL Protects ASS1-Deficient Cancers from Arginine Starvation–Induced Apoptosis

**DOI:** 10.1158/1078-0432.CCR-24-2548

**Published:** 2025-02-03

**Authors:** Prashanta Kumar Panda, Ana Carolina Paschoalini Mafra, Alliny C.S. Bastos, Li Cao, Maria Serra Bonet, Caitlyn B. Brashears, Ethan Yang Chen, Heather M. Benedict-Hamilton, William Ehrhardt, John Bomalaski, Carina Dehner, Leonard C. Rogers, Toshinao Oyama, Brian A. Van Tine

**Affiliations:** 1 Division of Medical Oncology, Department of Medicine, School of Medicine, Washington University in St. Louis, St. Louis, Missouri.; 2 Department of Orthopaedic, Union Hospital, Tongji Medical College, Huazhong University of Science and Technology, Wuhan, People’s Republic of China.; 3 Polaris Pharmaceuticals, Inc., San Diego, California.; 4 Department of Pathology and Laboratory Medicine, Indiana University School of Medicine, Indianapolis, Indiana.; 5 Siteman Cancer Center, St. Louis, Missouri.; 6 Department of Pediatric Hematology/Oncology, St. Louis Children’s Hospital, St. Louis, Missouri.

## Abstract

**Purpose:**

Argininosuccinate synthetase 1 (*ASS1*) silencing in carcinomas and sarcomas leads to a dependence on extracellular arginine for survival. Arginine deprivation therapies, such as PEGylated arginine deiminase (ADI-PEG20), have shown limited effectiveness, which may be due to underlying mechanisms that inhibit apoptosis.

**Experimental Design:**

The effects of ADI-PEG20 on cell-cycle regulation, apoptosis, and BCL-XL–mediated survival pathways in ASS1-deficient cancer cells were determined. The mechanism of cell death protection was determined by assessing caspase and PARP cleavage, CDK2 activity, MCL1 expression, and the interactions among BCL-XL, BAX, and BAK. *In vitro* synergy was determined, and *in vivo* efficacy was modeled.

**Results:**

Treatment with ADI-PEG20 led to reduced CDK2 activity and inhibited cell-cycle progression but did not induce significant cell death. BCL-XL was found to bind to BAX and BAK, preventing the initiation of apoptosis despite arginine starvation. Inhibition of BCL-XL allowed proapoptotic BAX and BAK to initiate the intrinsic apoptosis pathway, leading to increased cell death. This was found to be synergistic *in vitro* and efficacious in combination *in vivo*.

**Conclusions:**

The study identifies BCL-XL as a key factor limiting the efficacy of arginine starvation therapies. Combining BCL-XL inhibitors with arginine deprivation strategies may overcome this resistance and enhance therapeutic outcomes. These findings provide a strong preclinical rationale for testing this combination approach in phase 1 clinical trials for ASS1-deficient cancers.

**Translational Relevance**

The research highlights a critical metabolic adaptation in argininosuccinate synthetase 1–deficient cancers, which rely on external arginine for survival. Current therapies targeting arginine deprivation, such as PEGylated arginine deiminase, have demonstrated limited efficacy as monotherapies due to insufficient induction of cell death. This study reveals that the efficacy of these treatments is undermined by the protective role of BCL-XL, which binds to proapoptotic proteins and prevents apoptosis. Importantly, the combination of BCL-XL inhibitors with arginine starvation therapies, based on the observed mechanism in which BCL-XL inhibition allows proapoptotic BAX and BAK to trigger cell death, presents a promising strategy to enhance therapeutic outcomes. These findings provide a compelling preclinical basis for exploring the combination of BCL-XL inhibitors with PEGylated arginine deiminase in a clinical trial, potentially overcoming the limitations of current treatment approaches for argininosuccinate synthetase 1–deficient cancers.

## Introduction

The silencing of argininosuccinate synthetase 1 (*ASS1*) is a common occurrence in many solid and liquid tumors, as many sarcomas, melanomas, bladder cancers, prostate cancers, small cell lung cancers (SCLC), hepatocellular carcinomas, and leukemias are deficient in this enzyme (1–7). ASS1 is the rate-limiting enzyme in the urea cycle that converts L-citrulline to L-arginine (8). Arginine is considered a semiessential amino acid because it can be synthesized within a cell, as well as imported (8). Without the expression of ASS1, cancer cells become auxotrophic for arginine, resulting in a dependence on extracellular arginine sources for survival (8). The urea cycle is important, as many changes to the urea cycle have been seen in cancer and its microenvironment (9).

To harness arginine auxotrophy as a metabolic therapeutic approach for cancer, arginine deiminase, which catabolizes L-arginine back to L-citrulline and ammonia, has been PEGylated to form PEGylated arginine deiminase (ADI-PEG20; ref. 10). ADI-PEG20 is currently being tested in phase I to phase III clinical trials in many cancers. Although early studies of ADI-PEG20 as monotherapy were negative due to rapid metabolic adaptation, recent studies combining it with other treatments have shown positive outcomes (11–15). In addition, arginine starvation results in an increased uptake of nucleotides for biomass generation, which is also being harnessed in clinical trials for solid tumors (16).

In order to better understand the resistance mechanisms of metabolic adaptation to arginine starvation, canonical and noncanonical mechanisms of metabolic adaptation have been proposed (8, 17). The two classic canonical mechanisms of ADI failure are either for cancer to reexpress ASS1 in a Myc-dependent manner or through the formation of an antidrug antibody to ADI-PEG20 (16, 18–22). A newly described noncanonical pathway is via extracellular vesicular trafficking, a privileged site that is protected from extracellular arginine degradation (17). Noncanonical pathways are defined as prosurvival pathways that do not involve either ASS1 reexpression or the targeting of the enzyme itself by the immune system.

Early studies suggested that arginine starvation induced cell death via mitochondrial dysfunction or apoptosis (1, 4, 6, 23–29). This may be cancer subtype–specific, as bladder cancer may be more sensitive to arginine loss (6, 30). Careful examination of these early articles demonstrated that arginine starvation leads to cellular cytostasis with minimal cell death occurring in ASS1 low to nonexpressing cells. Additionally, low-level cell death from mitophagy has also been observed in breast cancer as an acute response to arginine starvation, but resistance to this mechanism also occurred rapidly, and the long-term ADI-treated cells adapted with upregulated mitochondrial function (22, 23). Therefore, it is unknown whether the lack of cell death resulting from arginine starvation is due to metabolic adaptation, mitochondrial alteration, or alterations in the apoptotic machinery in ASS1-deficient cancers.

Cancer cells often upregulate antiapoptotic mechanisms to evade cell death (31). The BCL-2 family of proteins are important regulators of the apoptotic intrinsic or mitochondrial pathway (32). Its main players are the proapoptotic or effector members BAX, BAK, and BOK and the prosurvival proteins BCL-2, BCL-XL, and MCL1. Frequently, enhanced expression and activity of antiapoptotic BCL-2 members are associated with cancer resistance to cell death by inhibiting BAX and BAK. BCL-XL has been described as one of the main regulators of BAX activity (33). Selective drugs designed to inhibit the BH3 domain of BCL-2 have been developed, such as venetoclax (34), but with little relevant activity seen as a single-agent therapy in solid tumors. Recent studies have described BCL-XL as the main regulator of BAX and that the disruption of the BCL-XL–BAX interaction leads to cell death by apoptosis (35).

Here, we demonstrate that arginine deprivation primes cells for apoptosis, inhibiting MCL1 activity through cell-cycle pause via CDK2 inhibition, but fails to induce apoptosis due to the activity of BCL-XL alone. With the addition of a BCL-XL selective inhibitor, A1331852, cell death increased *in vitro* (up to 90%), and a correspondingly significant reduction of tumor growth was observed *in vivo*. The success of this combination therapy both *in vitro* and *in vivo* sheds light on another noncanonical mechanism of arginine starvation–induced cell death and brings a promising clinical approach for the use of ADI-PEG20 treatment in patients.

## Materials and Methods

### Cell culture

All cell lines were maintained in a cell culture incubator with 5% CO_2_ at 37°C. SKLMS1 (ATCC, RRID:CVCL_0628), SKMEL2 (ATCC, RRID:CVCL_0069), and SKUT1 (ATCC, RRID:CVCL_0533) cells were grown in minimum essential medium (Gibco, #11095); RH28 [a gift from Lee J. Helman (USC Children’s Hospital, Los Angeles, California), RRID:CVCL_8752], LUPI [a gift from John Pfeifer (Washington University in St. Louis)], HT1080 (ATCC, RRID:CVCL_0317), DMS114 (ATCC, RRID:CVCL_1174), and NCI-H69AR (ATCC, RRID:CVCL_3513) were grown in RPMI 1640 medium (Gibco, #11875093); MG63 (ATCC, RRID:CVCL_0426), HTB93 (ATCC, RRID:CVCL_1734), MDAMB231 (ATCC, RRID:CVCL_0062), and MDAMB468 (ATCC, RRID:CVCL_0419) were grown in DMEM (Gibco, #11965084); and BVM02R was grown in Iscove’s modified Dulbecco’s medium (IMDM; Gibco, #12440046). All media, except Iscove’s modified Dulbecco’s medium, were supplemented with 10% FBS, which was supplemented with 20% FBS (Bio-Techne, #S11150), 1% 100X (10,000 U/mL) penicillin-streptomycin (Gibco, #15140148), and 2.5 mg/mL Plasmocin mycoplasma prophylactic (InvivoGen, #ant-mpp).

For live-imaging experiments, phenol red–free minimum essential medium (Gibco, #51200038), DMEM (Gibco, #51200038), and RPMI 1640 (Gibco, #11835030) with the above supplementation were used. For arginine-depleted media, ADI-PEG20 (Polaris) was added at 1 mg/mL, and media were kept at 37°C for 24 hours before performing experiments. A maximum concentration of 10 μmol/L was used for IC_50_ analyses of different drug treatments. Cells were monitored for mycoplasma with the MycoAlert Mycoplasma Detection Kit (Lonza, # LT07-118).

### Immunoblotting

SKLMS1 cells of 1 × 10^5^ and SKMEL2 cells of 2 × 10^5^ were seeded in six-well plates. After 24 hours, cells were treated with 1 μg/mL ADI-PEG20, 1 μmol/L A1331852 (DC Chemicals, #DC9296), 1 μg/mL ADI-PEG20 + 1 μmol/L A1331852, or vehicle control. After 6 hours (SKLMS1) or 12 hours (SKMEL2), cells were lysed with cell lysis buffer (Cell Signaling Technology, #9803) with Halt Protease and Phosphatase Inhibitor Cocktail (Thermo Fisher Scientific, #78440). Cell lysates were analyzed using 12–230 kDa separation modules (ProteinSimple, #SM-W004) on a Western capillary electrophoresis system (ProteinSimple, RRID:SCR_026213) and the total protein detection kit (ProteinSimple, #DM-TP01) according to the manufacturer’s instructions. BCL-XL (Cell Signaling Technology, Cat. # 2764S, RRID:AB_2228008), BCL-2 (Cell Signaling Technology, Cat. # 4223S, RRID:AB_1903909), MCL1 (Santa Cruz Biotechnology, Cat. # sc-12756, RRID:AB_627915), Bim (Cell Signaling Technology, Cat. # 2933, RRID:AB_1030947), Puma (Cell Signaling Technology, Cat. # 4976, RRID:AB_2064551), BAX (Cell Signaling Technology, Cat. # 5023, RRID:AB_10557411), BAK (Cell Signaling Technology, Cat. # 12105, RRID:AB_2716685), VDAC1/Porin (Abcam, Cat. # ab15895, RRID:AB_2214787), cleaved caspase-3 (Asp175; Cell Signaling Technology, Cat. # 9661, RRID:AB_2341188), cleaved PARP (c-PARP; Asp214; Cell Signaling Technology, Cat. # 5625, RRID:AB_10699459), CDK2 (Santa Cruz Biotechnology, Cat. # sc-6248, RRID:AB_627238), ME1 (Sigma-Aldrich, Cat. # SAB4501853, RRID:AB_10745296), phospho-CDK2 (Thr160; Cell Signaling Technology, Cat. # 2561, RRID:AB_2078685), and phospho-Mcl-1 (Ser64; Cell Signaling Technology, Cat. # 13297, RRID:AB_2798173) antibodies were used in the study. Protein levels were determined using Compass software for Simple Western (RRID:SCR_022930), and each band of primary antibodies was normalized to total protein.

### BAX and CDK2 knockdown

SKLMS1 and SKMEL2 cells were seeded in 60 mm plates and grown to 70% to 90% confluence. Control siRNA (Santa Cruz Biotechnology, sc-37007) and siRNAs targeting BAX (siBAX, Santa Cruz Biotechnology, sc-29212) and siRNAs targeting CDK2 (Thermo Fisher Scientific, 4390824-S206) transfections were conducted using Lipofectamine 3000 (Invitrogen, #L3000001), following the manufacturer’s instructions. Knockdown was evaluated 72 hours after transfection by immunoblotting.

### Gene overexpression

Plasmids for CDK2 (plasmid #1884, RRID:Addgene_1884), MCL1 (plasmid #21605, RRID:Addgene_21605), MCL1-3A (plasmid #197458, RRID:Addgene_197458), and EV (plasmid #200458, RRID:Addgene_200458) overexpression were obtained from Addgene. The bacterial strains were inoculated into 100 mL LB liquid medium containing antibiotics and cultured overnight at 37°C and 200 rpm. Bacterial strains harboring each plasmid were inoculated into 100 mL of LB medium supplemented with the corresponding antibiotic (ampicillin, Fisher Scientific, #AAJ6097706, or kanamycin, Fisher Scientific, #AAJ1792406) and cultured overnight at 37°C and 200 rpm. Plasmid DNA was extracted from the culture using the E.Z.N.A. Plasmid DNA Maxi Kit (Omega Bio-tek, Inc., #D6922) following the manufacturer’s instructions. A total of 3 × 10^5^ SKLMS1 and SKMEL2 cells were seeded into six-well plates, and on the following day, 2.5 μg of plasmid DNA (CDK2, MCL1, MCL1-3A, or EV) was transfected into the cells using Lipofectamine 2000 (Thermo Fisher Scientific, #11668027) according to the manufacturer’s instructions. An equal amount of empty vector (plasmid #200458, RRID:Addgene_200458) was used as a control. Proteins were extracted 48 hours after transfection for immunoblotting analysis, or cells were trypsinized 10 hours after transfection and analyzed for cell death using the Sartorius Incucyte S3 Live-Cell Analysis System (RRID:SCR_023147).

### Mitochondrial isolation

For mitochondria isolation, pellets of 2 × 10^7^ cells were prepared after centrifuging harvested cell suspensions of SKLMS1. Mitochondria isolation was performed according to the kit following the manufacturer’s instructions (Thermo Fisher Scientific, #89874). For protein analysis, mitochondria pellets were lysed with 2% CHAPS in TBS and vortexed for 1 minute. Lysates were centrifuged at 13,000 × *g* for 2 minutes at 4°C, and the soluble fraction was collected. Mitochondria and cytosolic proteins were characterized by VDAC1 and ME1, respectively.

### Automated image acquisition

Real-time, live cell images were captured using the Incucyte ZOOM (Sartorius, RRID:SCR_019874) and Incucyte S3 Live-Cell Analysis System (Sartorius, RRID:SCR_023147) in a 96-well format. For nuclei detection, cells were transduced with Incucyte Nuclight Red Lentivirus (EF-1α, Puro; Sartorius, #4476). Puromycin at 3 μg/mL (Fisher Scientific, #A1113803) was used for stable cell selection. The fluorescent green cell-impermeant dye, YOYO-1 iodide (Thermo Fisher Scientific, #Y3601), was used for the detection of dead cells. Proliferation fold change was calculated as follows: (red nuclei at time point)/(initial red nuclei count). For cell death percentage calculations, the following formula was used: (green count)/(red count + green count) × 100. For cell-cycle experiments, the cell-cycle lentiviral probe fluorescent ubiquitination-based cell-cycle indicator (Sartorius, #4779) was used. Incucyte image analysis software was used for all Incucyte assays. Incucyte Caspase-3/7 Green Dye (Sartorius, #4440) was used for the detection and analysis of apoptosis.

### Bliss model analysis

Bliss model analysis was performed to assess potential drug synergy between ADI-PEG20 and A1331852. The two drugs were combined at seven concentrations. The cell death rate was collected for analysis after 24 hours of treatment. The SynergyFinder tool (https://synergyfinder.fimm.fi/, RRID:SCR_019318) was used to construct the Bliss model. According to the user guide, a Bliss score greater than 10 indicates the presence of drug synergy.

### IHC

Five-micrometer sections of formalin-fixed, paraffin-embedded tissue were affixed to slides and baked at 50°C for 1 hour before deparaffinization in xylenes (Sigma, #247642; 2 × 5 minutes), rehydration (2 × 3 minutes) through a series of ethanol (EtOH) changes [100%, EtOH (Fisher Scientific, #A4094), 95% EtOH, 70% EtOH, and water], and blocking of endogenous peroxidase in 3% hydrogen peroxide (Sigma, #H1009-5ML). Next, citrate-based antigen unmasking solution (Vector Laboratories, #H-3300-250) was applied under heat for 5 minutes, followed by blocking in 3% w/v BSA (Sigma, #A9418) in 1× PBS (Fisher Scientific, # BP3991). Primary antibody against Ki-67 (Abcam, ab15580, RRID:AB_443209), 1:1,000 in blocking buffer, was applied for 5 minutes, followed by washing with 1× TBS (Fisher Scientific, #BP2471-1) with 0.1% Tween 20 (Sigma, #PT949). Slides were then incubated for 30 minutes in anti-rabbit secondary antibody (1:1,000, Jackson ImmunoResearch, #211-032-171, RRID:AB_2339149). The tissue was counterstained for 45 seconds in hematoxylin. Then slides were washed in water and dehydrated to 100% EtOH. After clearing in xylenes, coverslips were mounted using Cytoseal 60 Mounting Medium (Epredia, #831016). Tissue was visualized using an Olympus BX51 fluorescence microscope (Olympus Life Science, RRID:SCR_018949).

### *In vivo* xenograft model

For mouse engraft experiments, 5 × 10^6^ BVM02R cells were engrafted subcutaneously into the right flank of 4- to 6-week-old female C57BL/6J mice (The Jackson Laboratory). Tumors were measured using a digital caliper (Fisher Scientific, #15077958), and treatments were initiated once the size reached 200 mm^3^. ADI-PEG20 (Polaris) was administered at 9.6 mg/kg intramuscularly every 3 days at a dose of 13 μL of 11 mg/mL ADI-PEG20. A1331852 (DC Chemicals, DC9296) was dissolved in 60% PHOSAL 50 PG (Lipoid, 78036), 27.5% polyethylene glycol 400 (MilliporeSigma, #PX1286B-2), 10% EtOH, and 2.5% DMSO (36). A1331852 was administered at 25 mg/kg per mouse via oral gavage in a total volume of 200 μL. Treatments continued for 20 days. Tumor volume was measured daily using the following formula: (length × width^2^)/2. Body weight was determined prior to administration of each dose. Mice were euthanized when tumors reached 2,000 mm^3^, when tumors became ulcerated, or when the target time point was reached. Mouse tumor tissues were preserved with 10% neutral buffered formalin and embedded paraffin for further analysis. Animal studies were approved by the Institutional Animal Care and Use Committee at Washington University in St. Louis.

### Immunoprecipitation

To detect BCL-XL and BAX interaction, 2 × 10^6^ cells were seeded in 150 mm plates, for both SKLMS1 and SKMEL2. After 24 hours, cells were treated with ADI-PEG20, A1331852 (1 μmol/L), ADI-PEG20 + A1331852 (1 μmol/L), or vehicle control for 6 hours for SKLMS1 or 12 hours for SKMEL2. Cells were collected using a cell scraper and lysed with CHAPS lysis buffer [150 mmol/L NaCl, 50 mmol/L HEPES (pH 7.4), 0.1% CHAPS, and 0.5 mmol/L DTT] with 1× Halt Protease and Phosphatase Inhibitor Cocktail (Thermo Fisher Scientific, #78420). For each treatment group, 1,000 μg of lysate was incubated with 50 μL Dynabeads Protein A (Thermo Fisher Scientific, #10006D) + 1 μg of anti–BCL-XL (Cell Signaling Technology, Cat. #2764S, RRID:AB_2228008) or 1 μg of anti-IgG (Invitrogen, #02-6300) as control. The immunoprecipitation reaction was performed following the Dynabeads Protein A Immunoprecipitation Kit (Thermo Fisher Scientific, #10006D) manufacturer’s instructions. After the reaction, samples were incubated with NuPAGE LDS Sample Buffer (Thermo Fisher Scientific, # NP0007) and boiled for 10 minutes at 70°C. Protein samples were separated by electrophoresis on 4% to 20% Mini-PROTEAN precast gels (Bio-Rad, #4561094), transferred to polyvinylidene difluoride membranes (MilliporeSigma, # IPVH00010), blocked with a solution of 0.5% nonfat dry milk in TBS with 0.1% Tween 20, and then subjected to immunoblotting. Appropriate primary antibodies (1:10,000 in blocking buffer) were incubated overnight at 4°C. Membranes were washed with TBS and then incubated with a secondary antibody (1:10,000) at room temperature for 1 hour. Images were acquired in a ChemiDoc Imaging System (Bio-Rad, RRID:SCR_019684), and band densitometry was performed using the Image Lab software (Bio-Rad, RRID:SCR_014210). The following antibodies were used to detect proteins on the membrane: BCL-XL (Cell Signaling Technology, #2764S, RRID:AB_2228008), BAX (Cell Signaling Technology, # 2772S, RRID:AB_10695870), IgG (Invitrogen, #02-6300, RRID:AB_2532949), GAPDH (Novus, #NB300-221, RRID:AB_10077627), goat anti-rabbit IgG (The Jackson Laboratory, 111-035-144, RRID:AB_2307391), and goat anti-mouse IgG (The Jackson Laboratory, 111-035-146).

### Duolink proximity ligation assay

In a 96-well glass bottom plate (Cellvis, #P96-1.5H-N), 2 × 10^4^ cells were seeded, for both SKLMS1 and SKMEL2. After 24 hours, cells were treated with ADI-PEG20 (1 μg/mL), A1331852 (1 μmol/L), ADI-PEG20 (1 μg/mL) +A1331852 (1 μmol/L), or vehicle control for 6 hours for SKLMS1 or 12 hours for SKMEL2. Cells were fixed using 4% paraformaldehyde (Thermo Fisher Scientific, #J19943.K2) and permeabilized with 0.5% Triton X-100 (MilliporeSigma, #1086431000), both for 15 minutes followed by 1× PBS washes. Blocking was performed for 60 minutes at 37°C using the Duolink Blocking Solution (1×) provided in the Duolink *In Situ* Detection Reagents Red kit (Thermo Fisher Scientific, #DUO92008-100RXN). Samples were then incubated with primary antibodies anti–BCL-XL mouse 1:200 (Thermo Fisher Scientific, Cat. # MA5-11950, RRID:AB_10986767) and anti–BAX (Cell Signaling Technology, #2772S, RRID:AB_10695870) overnight at 4°C. Controls were implemented for each combination of Proximity Ligation Assay (PLA) probes by excluding both primary antibodies. After this step, the Duolink Proximity Ligation Assay was conducted following the manufacturer’s instructions. Images were acquired using the microscope Celldiscoverer 7 ZEISS LSM 900 (ZEISS, RRID:SCR_022263) at the Washington University Center for Cellular Imaging (RRID:SCR_015140). Duolink Proximity Ligation Assay signals of the interaction were quantified using ImageJ software (NIH USA, RRID:SCR_003070), in which only the red channel corresponding to the PLA signal was used for analysis.

### Statistical analysis

All statistical tests were analyzed using GraphPad Prism 9 software (RRID:SCR_002798). All data are presented as mean ± SD. Experiments were performed with biological replicates. Grouped data were analyzed by ratio paired *t* test or unpaired *t* test as appropriate.

### Data availability

All data from this study are found in the present article or in supplementary sections. All unique/stable reagents generated in this study are available from the corresponding author with a completed material transfer agreement. All other data are available in the main text or the Supplementary Materials. This article does not report the original code. Any additional information required to reanalyze the data reported in this article is available from the corresponding author upon request. All further information and requests for resources and reagents should be directed to and will be fulfilled by the corresponding author.

## Results

### Arginine starvation with ADI-PEG20 inhibits proliferation through cell-cycle inhibition without initiating cell death in ASS1-deficient cancer cell lines

**Figure 1.**
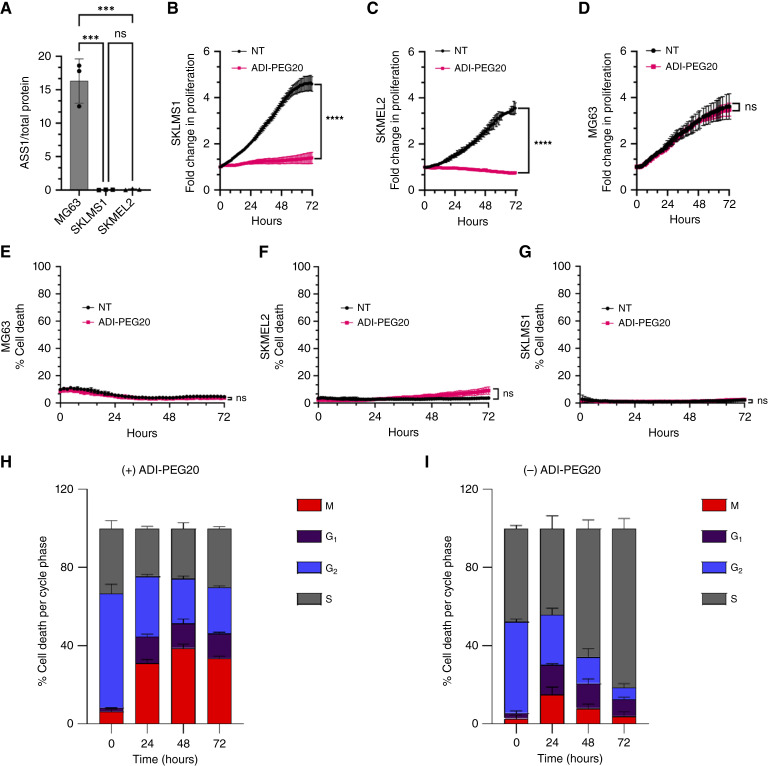
Arginine starvation induces cell-cycle arrest in ASS1-null cancer cell lines *in vitro*. **A,** ASS1 protein expression in MG63, SKLMS1, and SKMEL2 cells. **B–D,** Normalized proliferation of SKLMS1, SKMEL2, and MG63 cells with and without ADI-PEG20 treatment *in vitro*, respectively. **E–G,** Percentage of cell death of SKLMS1, SKMEL2, and MG63 cells with and without ADI-PEG20 treatment *in vitro*, respectively. **H** and **I,** Percentage of cells in each cell-cycle phase (G_1_, S, G_2_, and M) at 0, 24, 48, and 72 hours in SKLMS1 cells with and without ADI-PEG20 treatment *in vitro*, respectively. Data are mean ± SD (*n* = 3). One-tailed paired *t* tests were used for **A–G**. ***, *P* < 0.001; **** *P *< 0.0001.

To investigate the effects of arginine starvation in ASS1-deficient cancers, the ASS1-null leiomyosarcoma cell line SKLMS1 and the melanoma cell line SKMEL2 were used, whereas the osteosarcoma cell line MG63 was used as an example of a high expresser of ASS1 (Fig. 1A; refs. 1, 22, 29). Arginine starvation induced by ADI-PEG20 led to a static growth rate in SKLMS1 and SKMEL2 but did not affect the proliferation of MG63 (Fig. 1B–D). In addition, significant cell death was not observed in any cell line over 3 days with ADI-PEG20 treatment (Fig. 1E–G).

To determine if the static growth rate was due to a cell-cycle checkpoint arrest, the percentage of cells in each cell-cycle phase was analyzed over 72 hours using the Incucyte lentiviral fluorescent ubiquitination-based cell-cycle indicator. ADI-PEG20 prevented cells from transitioning among cell-cycle phases, demonstrating a generalized cell-cycle pause related to where cells were at the beginning of ADI-PEG20 treatment (Fig. 1H; Supplementary Fig. S1A). Nontreated control cells continued to transition through cell-cycle phases until reaching confluence, in which an accumulation in the S-phase was seen (Fig. 1I; Supplementary Fig. S1B).

### ADI-PEG20 sensitizes cells to apoptosis by BCL-XL inhibition

**Figure 2.**
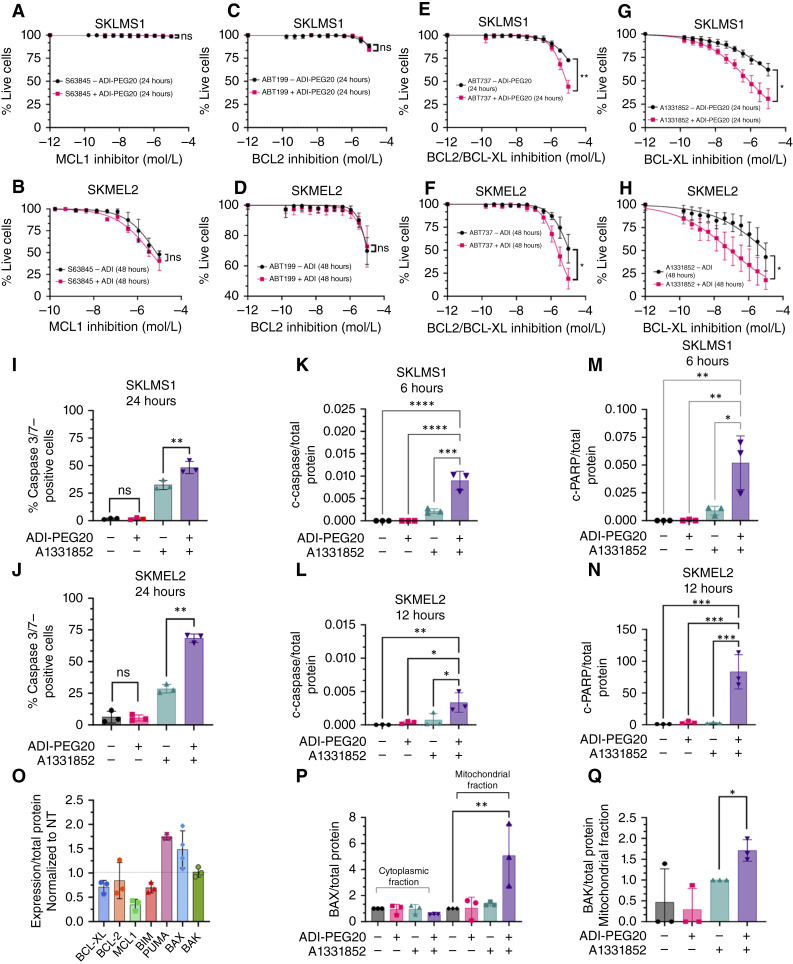
**A–H,** ADI-PEG20 treatment primes cells for cell death via BCL-XL inhibition. Percentage of live cells was determined for SKLMS1 cells (**A**) and SKMEL2 cells (**B**) treated with MCL1 inhibitor S63845 with/without ADI-PEG20 *in vitro*, SKLMS1 (**C**) and SKMEL2 cells (**D**) treated with BCL2 inhibitor ABT199 with/without ADI-PEG20, SKLMS1 (**E**) and SKMEL2 cells (**F**) treated with BCL2/ BCL-XL inhibitor ABT737 with/without ADI-PEG20, and SKLMS1 (**G**) and SKMEL2 (**H**) treated with BCL-XL inhibitor A1331852 with/without ADI-PEG20. **I** and **J,** Percentage of positive caspase 3/7 cells after 24 hours of treatment with or without ADI-PEG20 and A1331852 in SKLMS1 and SKMEL2, respectively. **K** and **L,** Protein expression of cleaved caspase (c-caspase) after 6 hours in SKLMS1 cells and after 12 hours in SKMEL2 cells, with/without ADI-PEG20 and A1331852 treatment, respectively. **M** and **N,** Protein expression of c-PARP after 6 hours in SKLMS1 cells and after 12 hours in SKMEL2 cells, with/without ADI-PEG20 and A1331852 treatment, respectively. **O,** Expression of apoptosis-related proteins in SKLMS1 cells with ADI-PEG20 treatment. **P,** BAX protein expression in cytoplasmic and mitochondrial fractions of SKLMS1 cells with/without ADI-PEG20 and A1331852 treatment. **Q,** BAK protein expression in mitochondrial fraction of SKLMS1 cells with/without ADI-PEG20 and A1331852 treatment. Data are mean ± SD (*n* = 3). Two-tailed paired *t* tests were used for **A–N**, **P**, and **Q**. *, *P* < 0.05; **, *P* < 0.01; ***, *P* < 0.001; ****, *P* < 0.0001.

Given that growth inhibition, but not cell death, was observed following ADI-PEG20 treatment of cells, the effect of arginine starvation on apoptotic pathways was directly investigated. First, the activity of several antiapoptotic proteins of the BCL-2 family (BCL-2, MCL1, and BCL-XL) under arginine starvation was examined using pharmacologic inhibition. ADI-PEG20, in combination with MCL1 inhibitor (S58345), did not sensitize ASS1-null SKLMS1 and SKMEL2 cells to cell death (Fig. 2A and B).

Likewise, treatment with ADI-PEG20, when combined with BCL-2 inhibitor ABT199, did not sensitize cells to cell death (Fig. 2C and D). Although some effect was seen with dual BCL-2/BCL-XL inhibitor ABT737 at high doses (Fig. 2E and F), treatment with the specific BCL-XL inhibitor A1331852, when combined with ADI-PEG20, promoted cell death in a dose-dependent manner (Fig. 2G and H). Cell death was found to initiate 6 hours earlier in SKLMS1 cells than in SKMEL2 cells.

Additionally, cleaved caspase, c-PARP, and caspase 3/7 expression levels were measured. The percentage of caspase 3/7–positive cells increased following ADI-PEG20 treatment in combination with A1331852 as compared with BCL-XL inhibition alone (Fig. 2I and J). Along with that, elevated levels of cleaved caspase and c-PARP were observed following combination treatment compared with untreated controls (Fig. 2K–N). Together, these results demonstrate that AD-PEG20 is altering the balance of pro- and antiapoptotic proteins but is not able to induce apoptosis alone.

### BCL-XL inhibition enhances BAX and BAK mitochondrial localization in arginine-starved cells

Next, to determine the effect of ADI-PEG20 treatment on apoptosis-related proteins, the level of expression of multiple BCL-2 family members was measured by whole-exome sequencing. Expression levels of BCL-XL, MCL1, and Bim decreased significantly following ADI-PEG20 treatment (Fig. 2O). The BH3-only proteins PUMA and BAX increased (Fig. 2O). The mitochondrial membrane pore–forming protein BAK was unchanged (Fig. 2O).

As BCL-XL binds to BAX and BAK (35), preventing BAX and BAK localization to the mitochondria in which they participate as downstream effectors of the apoptotic cascade, mitochondrial localization of BAX and BAK was investigated under ADI-PEG20 treatment alone or in combination with Bcl-xL inhibitor A1331852. Combination treatment of ADI-PEG20 and A1331852 increased the expression of both BAX and BAK in the mitochondrial fraction (Fig. 2P and Q).

Moreover, arginine starvation induced by ADI-PEG20 moderately disrupted the interaction between BCL-XL and BAX, as shown via co-immunoprecipitation and immunoblot analysis (Supplementary Fig. S2A and S2B). Combined treatment with ADI-PEG20 and A1331852, or A1331852 alone, led to a further decrease in BAX–BCL-XL interaction, as demonstrated by reductions in both PLA signals and BAX band density (Supplementary Fig. S2C–S2E). Together, these results suggest that ADI-PEG20 treatment disrupts the BCL-XL–BAX interaction, promoting BAX and BAK mitochondrial localization and enhancing the potential for apoptosis when combined with BCL-XL inhibition.

### ADI-PEG20 treatment downregulates the antiapoptotic protein MCL1 through CDK2 inhibition

**Figure 3.**
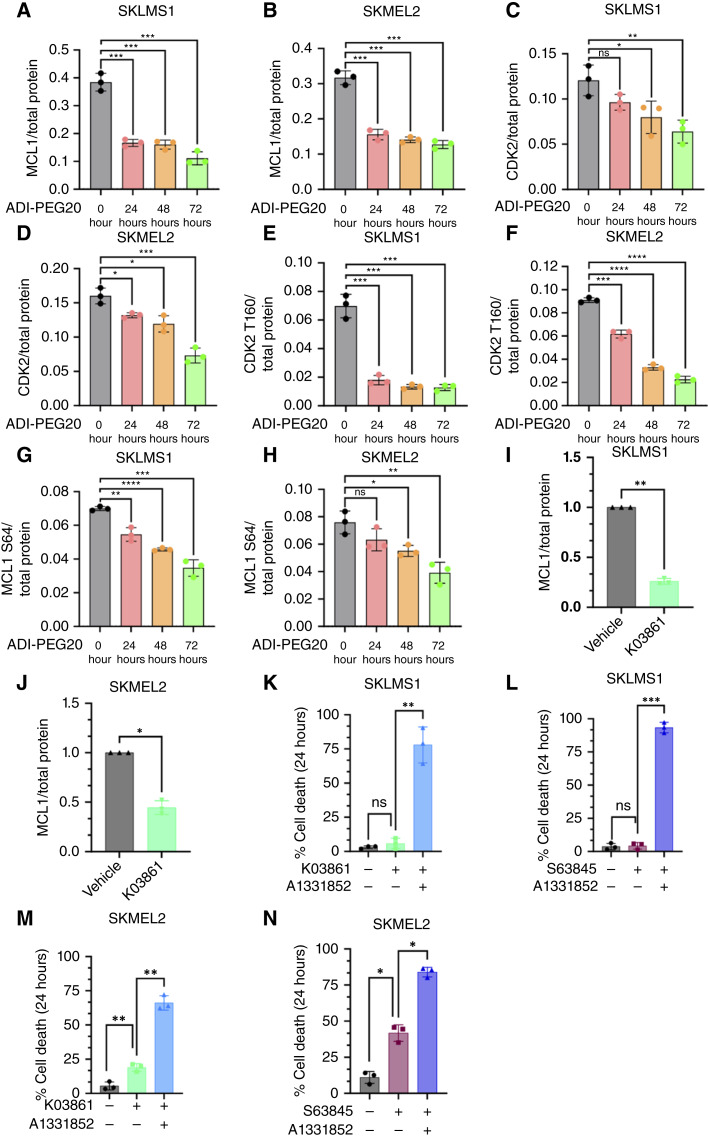
Arginine starvation downregulates MCL1 through CDK2 inhibition. **A** and **B,** MCL1 expression in 1 μg/mL ADI-PEG20 for 0, 24, 48, and 72 hours in SKLMS1 and SKMEL2 cells, respectively. **C** and **D,** CDK2 expression in ADI-PEG20 for 0, 24, 48, and 72 hours in SKLMS1 and SKMEL2 cells, respectively. **E** and **F,** The phosphorylation of CDK2 T160 in ADI-PEG20 for 0, 24, 48, and 72 hours in SKLMS1 and SKMEL2 cells, respectively. **G** and **H,** The phosphorylation of MCL1 S64 in ADI-PEG20 for 0, 24, 48, and 72 hours in SKLMS1 and SKMEL2 cells, respectively. **I** and **J,** MCL1 expression in untreated (NT) vs. 3 μmol/L CDK2 inhibitor K03861 treatment in SKLMS1 and SKMEL2 cells, respectively. **K** and **L,** Percentage of cell death at 24 hours in SKLMS1 cells left untreated, treated with 3 μmol/L CDK2 inhibitor (K03861), or treated with CDK2 inhibitor in combination with 1 μmol/L BCL-XL inhibitor (A1331852). **M** and **N,** Percentage of cell death after 24 hours in SKMEL2 cells left untreated, treated with 1 μmol/L MCL1 inhibitor (S63845), or treated with a combination treatment of 1 μmol/L BCL-XL inhibitor (A1331852) and 1 μmol/L MCL1 inhibitor (S63845). Data are mean ± SD (*n* = 3). One-tailed paired *t* tests were used for **I** and **J**. Two-tailed paired *t* tests were used for **A–H** and **K–N**. *, *P* < 0.05; **, *P* < 0.01; ***, *P* < 0.001; ****, *P* < 0.0001.

As MCL1 inhibition alone following ADI-PEG20 treatment did not induce cell death (Fig. 2A and B), the suspected downregulation of MCL1 in SKLMS1 and SKMEL2 cells was confirmed via immunoblot (Fig. 3A and B). Given that arginine starvation induced by ADI-PEG20 causes global cell-cycle effects, the expression level of CDK2, which regulates the expression of MCL1 by preventing proteasomal degradation, was measured (37–39). CDK2 expression and its phosphorylation of T160 were decreased with ADI-PEG20 treatment in SKLMS1 and SKMEL2 cells (Fig. 3C–F). As expected, over the course of 72 hours of ADI-PEG20 exposure, ASS1 reexpression was also observed, a known mechanism by which ASS1-null cells have to adapt to arginine starvation via ADI-PEG20 treatment (Supplementary Fig. S3A and S3B).

Changes in MCL1 phosphorylation at serine 64 (S64), a phosphorylation site known to be targeted by CDK2 (37–39), following ADI-PEG20 treatment were further investigated. Prolonged ADI-PEG20 treatment resulted in a reduction of both total MCL1 and MCL1 phosphorylated at S64 (Fig. 3G and H), indicating that MCL1 depletion is an indirect effect of arginine starvation caused by the lack of CDK2.

To confirm that arginine starvation depletes MCL1 through CDK2 inhibition, SKLMS1 and SKMEL2 cells were treated with the CDK2 inhibitor K03861. As expected, MCL1 levels were decreased (Fig. 3I and J) under this condition, thus linking the decrease in MCL1 to the cell-cycle effect of ADI-PEG20 in repressing CDK2 expression.

### MCL1 downregulation through CDK2 sensitizes cells to BCL-XL inhibition

Concomitant inhibition of BCL-XL and CDK2 or MCL1 increased cell death after 24 hours of treatment in both SKMLS1 and SKMEL2 cells (Fig. 3K–N). This indicates that MCL1 downregulation through CDK2 inhibition sensitizes cells to inhibition by BCL-XL.

To verify that BCL-XL protected cells from cell death through its interaction with BAX, we generated *BAX* knockdowns using si*BAX* in SKLMS1 and performed cell death experiments with combination treatment. Significant protection from cell death and PARP cleavage was observed using si*BAX* after arginine starvation and BCL-XL inhibition (Supplementary Fig. S3C and S3D).

Combined, these results support the hypothesis that MCL1 downregulation through CDK2 inhibition via arginine starvation sensitizes to BCL-XL inhibition.

### Arginine starvation–induced repression of MCL1 is driven by CDK2

**Figure 4.**
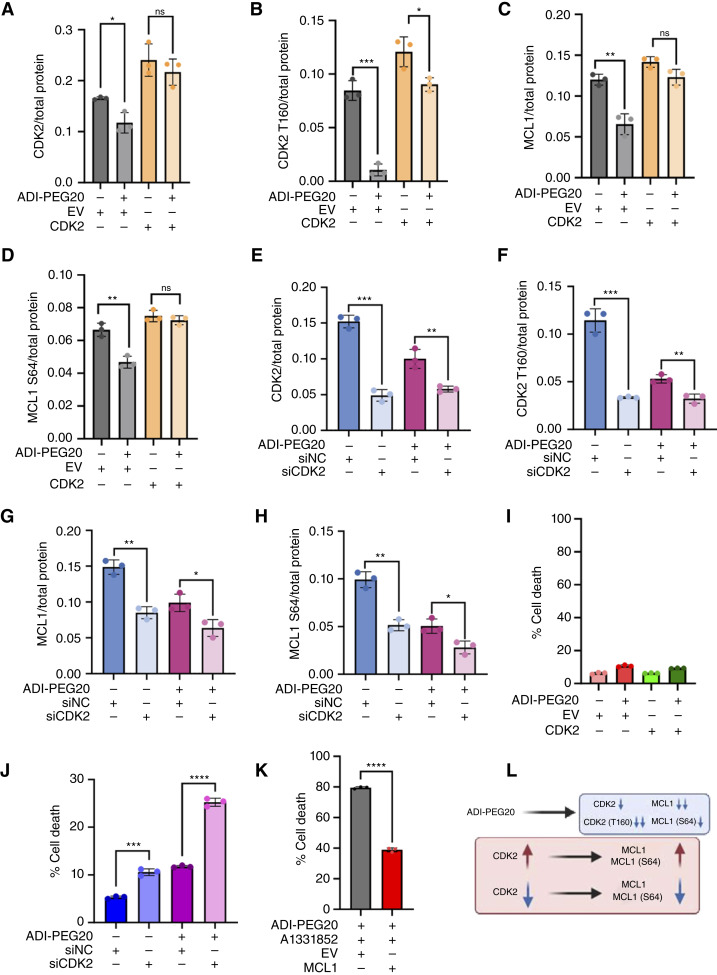
CDK2 mediates the repression of MCL1 during arginine starvation. **A–D,** CDK2 protein expression (**A**), CDK2 T160 phosphorylation (**B**), MCL1 protein expression (**C**), and MCL1 S64 phosphorylation (**D**) in SKLMS1 cells overexpressed CDK2 or empty vector (EV) and subsequently treated with ADI-PEG20. **E–H,** CDK2 protein expression (**E**), CDK2 T160 phosphorylation (**F**), MCL1 protein expression (**G**), and phosphorylation of MCL1 S64 (**H**) in SKLMS1 cells knocked down CDK2 or nontargeting control (NC) and subsequently treated with ADI-PEG20. **I,** Percentage of cell death of CDK2 overexpressed SKLMS1 cells with ADI-PEG20 treatment at 24 hours. **J,** Percentage of cell death of CDK2 knocked down SKLMS1 cells with ADI-PEG20 treatment at 24 hours. **K,** Percentage of cell death of MCL1 overexpressed SKLMS1 cells with combined treatment of ADI-PEG20 and A1331852 at 24 hours. **L,** Schematic illustration of MCL1 inhibition caused by ADI-PEG20 through CDK2 inhibition. Two-tailed paired *t* tests were used for **A–K**. *, *P* < 0.05; **, *P* < 0.01; ***, *P* < 0.001; ****, *P* < 0.0001. (**L**, Created with BioRender.com. Li, C. [2025], https://BioRender.com/h63u462.)

To confirm that MCL1 repression by ADI-PEG20 treatment occurs through CDK2 repression, CDK2 was overexpressed in SKLMS1 and SKMEL2 cells (Fig. 4A and B; Supplementary Fig. S4A and S4B). The overexpression of CDK2 restored MCL1 protein levels and its phosphorylation at S64 during ADI-PEG20 treatment (Fig. 4C and D; Supplementary Fig. S4C and S4D). Additionally, CDK2 knockdown (Fig. 4E and F; Supplementary Fig. S4E and S4F) significantly repressed MCL1 protein expression and its phosphorylation at S64 under untreated conditions but only slightly repressed MCL1 under ADI-PEG20 treatment (Fig. 4G and H; Supplementary Fig. S4G and S4H), and CDK2 overexpression did not alter baseline cell death after treatment with ADI-PEG20 (Fig. 4I; Supplementary Fig. S4I). Finally, CDK2 knockdown under ADI-PEG20 treatment did not show a significant difference in cell death induction compared with the untreated condition (Fig. 4J; Supplementary Fig. S4J).

In agreement, overexpression of MCL1 also protected cells from the combination treatment of ADI-PEG20 and A-1331852, leading to a reduction in cell death (Fig. 4K; Supplementary Fig. S4K–S4O). Collectively, these findings demonstrate that the loss of MCL1 and its antiapoptotic function, driven by the repression of CDK2 under arginine starvation, plays a significant role in promoting cell death in ASS1-deficient cells.

### ADI-PEG20 and A1331852 synergize to induce cell death in ASS1-deficient cancer cell lines

**Figure 5.**
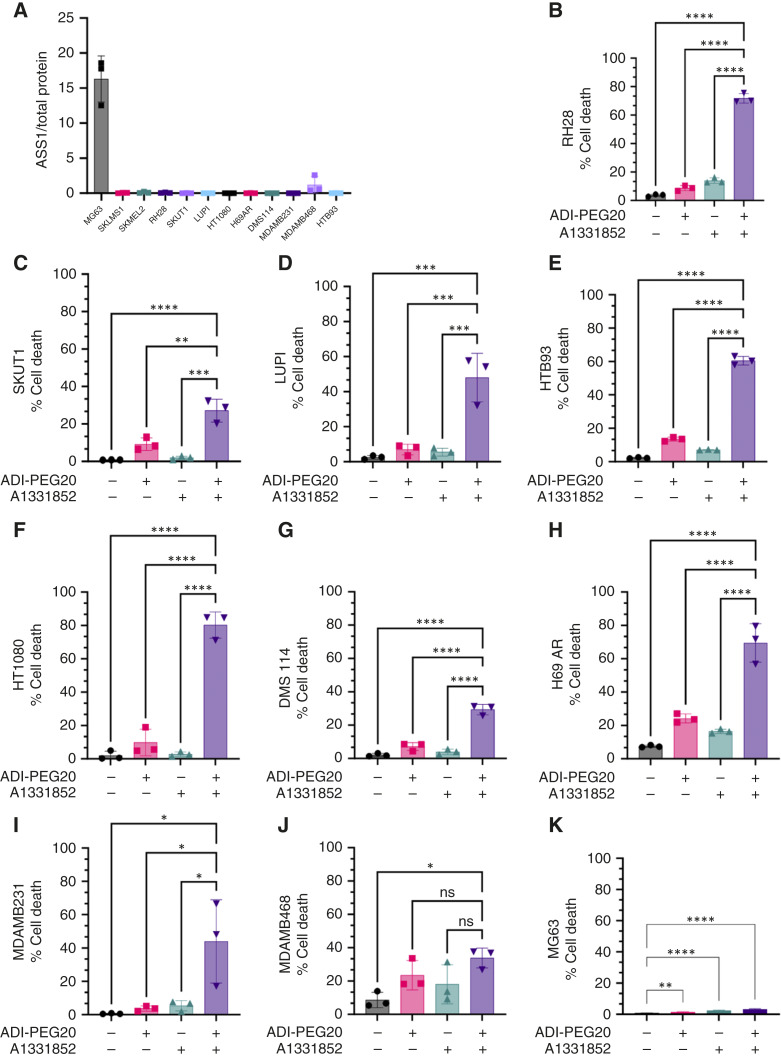
Combination treatment of ADI-PEG20 and A1331852 induces cell death in ASS1-deficient cancer cell lines. **A,** ASS1 protein expression in MG63, SKLMS1, SKMEL2, RH28, SKUT1, LUPI, HT1080, H69AR, DMS 114, MDAMB231, MDAMB468, and HTB93 cells. **B–K,** Percentage of cell death upon ADI-PEG20, A1331852, or combination treatment after 48 hours in RH28, SKUT1, LUPI, HTB93, HT1080, DMS 114, H69AR, MDAMB231, MDAMB468, and MG63 cells, respectively. Data are mean ± SD (*n* = 3). Two-tailed paired *t* tests were used for **B–K**. *, *P* < 0.05; **, *P* < 0.01; ***, *P* < 0.001; ****, *P* < 0.0001.

The percentage of cell death following ADI-PEG20–induced arginine starvation was evaluated in a panel of cell lines from different solid tumors with differing levels of ASS1 to validate that BCL-XL rescues cells from apoptosis according to ASS1 expression status. Dual treatment with ADI-PEG20 and A1331852 increased the percentage of cell death in cell lines originating from sarcomas with no or little ASS1 expression, such as RH28, SKUT1, LUPI, HTB93, and HT1080 (Fig. 5A–F). Cell death increase was also observed in other ASS1-null cancer cells within the panel tested, including the SCLC cell lines DMS 114 and H69 AR (Fig. 5A, G, and H) and the breast cancer cell line MDAMB231 (Fig. 5A and I). ASS1-expressing cells, such as the breast cancer cell line MDAMB468 (Fig. 5A and J) or the osteosarcoma cell line MG63, did not respond in a meaningful way to dual treatment with ADI-PEG20 and A1331852 (Fig. 5A and K).

In addition, a synergy assay with different concentration gradients for ADI-PEG20 and A1331852 suggested that the combination of the two drugs showed a certain synergistic effect in SKLMS1 and SKMEL2 cells (Supplementary Fig. S5A and S5B).

### Arginine starvation and BCL-XL combination treatment markedly reduces tumor growth and cell proliferation *in vivo*

**Figure 6.**
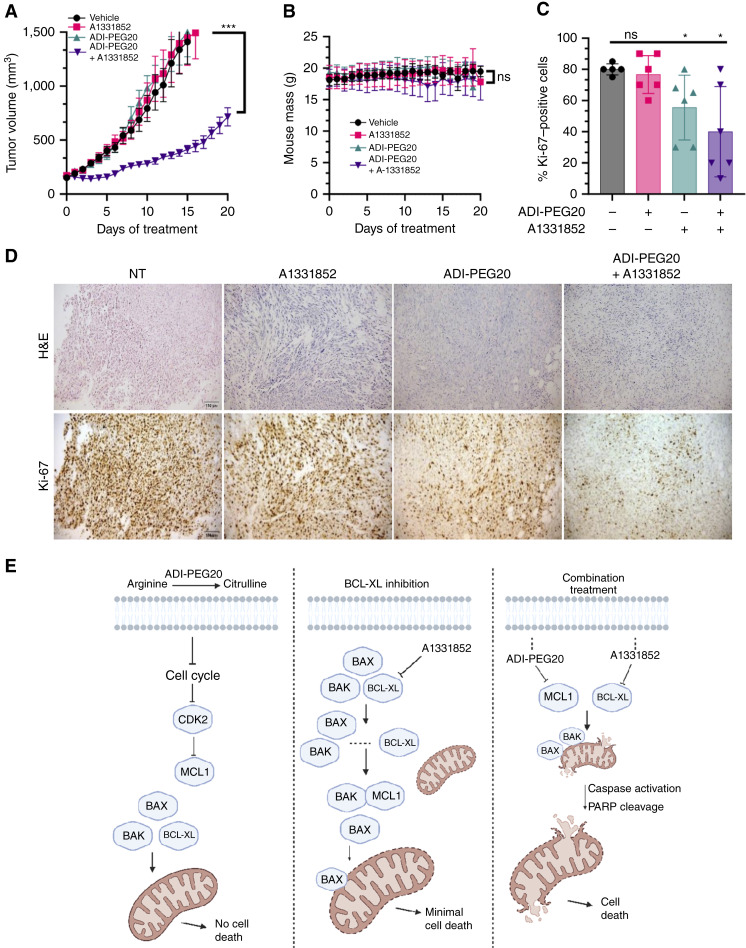
ADI-PEG20 and A1331852 treatment reduces tumor growth *in vivo*. **A,** Volumes of BVM02R tumors in syngeneic C57Bl/6J mice treated with vehicle, ADI-PEG20, A1331852, or the combination of both. **B,** Weights of mice from **A**. **C,** Percentage of positive Ki-67 cells with or without both ADI-PEG20 and A1331852 treatments. **D,** Representative images of hematoxylin and eosin (H&E) and IHC stains of Ki-67 analysis of mice from **A**. **E,** Schematic representation of herein proposed mechanism, combining MCL1 inhibition through cell-cycle pause via CDK2 inhibition caused by arginine starvation and BCL-XL inhibition to induce tumor cell death. Data are mean ± SD (*n* = 6 animals per group). Two-way ANOVA was used for **A** and **B**. Two-tailed paired *t* tests were used for **C**. *, *P* < 0.05; ***, *P* < 0.001. (**E**, Created with BioRender.com. Hirbe, A. [2025], https://BioRender.com/e14u849.)

C57Bl/6J mice were injected with a syngeneic rhabdomyosarcoma cell line (BVM02R; ref. 17) and then treated for 20 days with either ADI-PEG20 or A1331852 or in combination, versus control (vehicle). Although nonsyngeneic cell lines demonstrate growth inhibition following ADI-PEG20 treatment in immunocompromised mice, syngeneic models do not due to arginine being provided via noncanonical vesicular trafficking (17, 40). Unlike *in vitro* studies, single-agent ADI-PEG20 treatment did not have a significant effect on cell growth *in vivo* when a syngeneic system was used (Fig. 6A). In addition, the BCL-XL inhibitor had no significant effect on tumor growth (Fig. 6A). However, combination treatment of arginine starvation and BCL-XL inhibition reduced tumor growth significantly (Fig. 6A). Mouse body weight was measured throughout the experiment, and no significant change was observed for any of the treatment groups (Fig. 6B). The decrease in the tumor growth observed *in vivo* was supported by Ki-67 IHC staining of tumor tissue (Fig. 6C and D). These results confirm the synergistic efficacy of combined arginine starvation and BCL-XL inhibition treatment *in vivo*.

## Discussion

The development of arginine deprivation strategies has been limited by an incomplete understanding of the canonical and noncanonical mechanisms of adaptation to arginine starvation. Further understanding of these mechanisms is needed if metabolic therapies are to be translated into clinical practice. Here, we define another noncanonical mechanism, whereby BCL-XL acts as a prosurvival signal and prevents cell death during the acute phase of arginine starvation. Although the canonical mechanism of reexpressing ASS1 requires time and multiple steps including demethylation, likely over several cell divisions, and immune clearance of ADI-PEG20 via anti-ADI antibodies occurs over the course of several weeks, this newly defined noncanonical BCL-XL prosurvival pathway is likely one of the first pathways initiated to prevent cell death in the arginine starvation state. The subsequent bypassing of extracellular arginine deprivation through the utilization of extracellular vesicular trafficking from the microenvironment would likely occur (17).

Although ADI-PEG20 as a single-agent therapy does not cause cell death, this study demonstrates that by inhibiting the prosurvival apoptotic protein BCL-XL, cell death can be achieved in the arginine starvation state. The loss of cellular proliferation caused by ADI-PEG20 causes an indirect MCL1 loss that occurs via CDK2 inhibition (Fig. 3A–J), thus leaving BCL-XL to act as a prosurvival pathway. When it is inhibited with A1331852 without arginine deprivation, the disruption of BCL-XL–BAX interactions occurs, but MCL1 binds to BAK and impairs the ability to initiate cell death. Combination treatment thus impairs both BCL-XL and MCL1 regulatory activities at the same time, allowing for BAX and BAK to localize to the mitochondria, activate the apoptotic cascade, and induce cell death (Fig. 6E). This allows for the efficient prosurvival adaptation of ASS1 nonexpressing tumors.

The major utility of this discovery is that it advances our understanding of how cells survive while metabolically adapting to live in a state of arginine starvation. The initial antiapoptotic response to arginine starvation allows for tumors to survive, whereas they adapt to an extracellular environment in which arginine is absent. This is accomplished via either ASS1 upregulation or support from the microvesicular trafficking that is privileged from extracellular arginine depletion (16). In addition, to survive, the response to arginine starvation is rapid and results in a switch in cells from a Warburgian biology to an oxidative biology and glutamine dependence for survival (22). It is in this situation that glutaminase inhibitors have been found to be cytotoxic. In addition, cells are also starved for nucleotides and upregulate transporters for the importation of biomass (15). These findings are already being tested in clinical trials.

Resistance to ADI-PEG20 can arise through both canonical and noncanonical mechanisms, which represents a challenge for clinical development. The approach currently being taken involves drugging the metabolic adaptation to ADI-PEG20. First, gemcitabine with docetaxel has been demonstrated to prolong the appearance of autoantibodies in the sarcoma clinical trial (data unpublished). Second, instead of preventing ASS1 expression, the ADI-forced expression forces a metabolic reprogramming that allows cells to overcome hENT receptor desensitization (16, 22). In addition, to address the noncanonical mechanisms, we have shown that the addition of chloroquine overcomes extracellular vesicular trafficking that can provide an arginine source and that glutaminase inhibition causes cell death (1, 16, 22). Finally, to add to noncanonical mechanisms, the antiapoptotic strategy that ASS1-low cells use to prevent cell death has been identified. Which and how many of these mechanisms can be addressed at the same time await formal testing in phase I clinical trials. Although ADI-PEG20 with gemcitabine and docetaxel is currently in a registration trial, a phase I trial adding chloroquine to the combination is under development.

BCL-XL inhibition with small molecule inhibitors has been associated with significant challenges, including thrombocytopenia and other toxicities, which have limited its clinical application (36). These adverse effects primarily arise from the dependence of platelets on BCL-XL for survival, as BCL-XL is crucial for maintaining platelet homeostasis (41). Emerging approaches, such as the development of proteolysis-targeting chimera (PROTAC) degraders like DT2216, represent promising alternatives to circumvent these limitations. DT2216 selectively targets BCL-XL for degradation in tumor cells while sparing platelets, potentially reducing the risk of thrombocytopenia (42). Preclinical studies have demonstrated its efficacy in various cancer models with a significantly improved toxicity profile. However, these strategies are still in the early stages of development, and their clinical feasibility, long-term safety, and effectiveness require further validation.

Our finding that ASS1-negative cells become reliant on BCL-XL represents a therapeutic opportunity for combining ADI-PEG20 with a BCL-XL inhibitor that may be added to current therapies. Although in our model A1331852 was utilized, the PROTACs of BCL-XL that are in early clinical development may allow for true clinical translation of this vulnerability (43) and are in a phase I clinical trial (NCT04886622). Whether this needs to be combined with additional agents such as the ADI-PEG20, gemcitabine, and docetaxel combinations being tested in sarcoma, non–small cell lung cancer, and SCLC or if it can be used as a standalone combination therapy with ADI-PEG20 awaits formal testing in clinical trials.

## Supplementary Material

Supplementary Figure S1 ADI-PEG20 treatment causes cell cycle pause

Supplementary Figure S2 ADI-PEG20 and A1331852 combination treatment disrupts BCL-XL:BAX interaction in vitro

Supplementary Figure S3 BAX knockdown represses cell death induced by the combined treatment of ADI-PEG20 and A1331852

Supplementary Figure S4 Arginine starvation leads to MCL1 repression through CDK2 repression

Supplementary Figure S5 ADI-PEG20 and A1331852 synergically induce cell death.
