# Supplementary material for: BCL-XL Protects ASS1-Deficient Cancers from Arginine Starvation–Induced Apoptosis
Source: Clin Cancer Res. 2025 Feb 3;31(7):1333–45. doi: 10.1158/1078-0432.CCR-24-2548 (PMC11964295; doi:10.1158/1078-0432.CCR-24-2548)
Supplement: Supplementary Figure S1 — ADI-PEG20 treatment causes cell cycle pause [file ccr-24-2548_supplementary_figure_s1_suppfs1.pdf]

SUPPLEMENTARY FIGURE 1

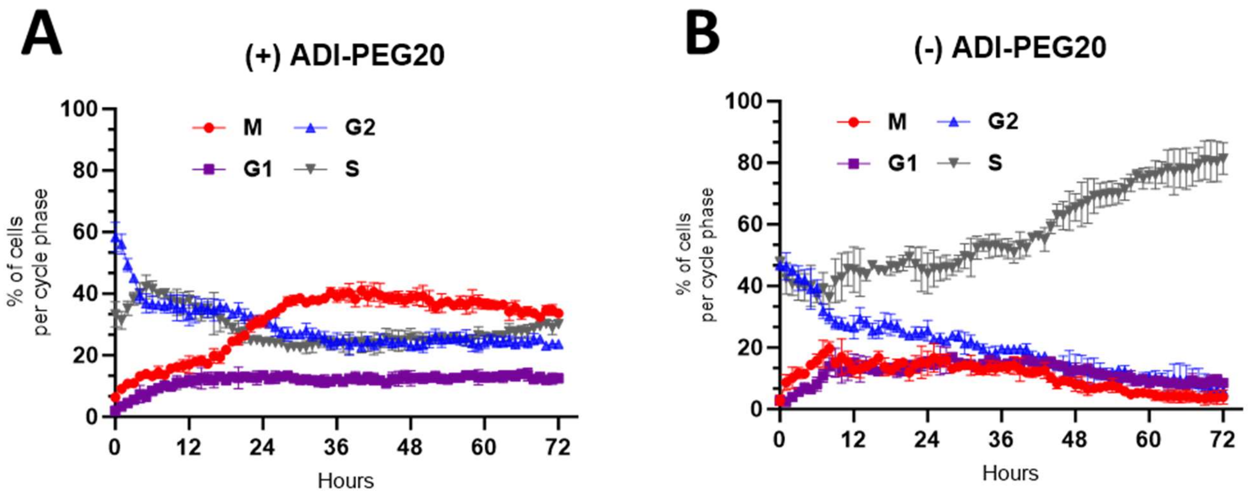

Supplementary Figure 1.

ADI-PEG20 treatment causes cell cycle pause. **A, B,** Percentage of cells in each cell cycle phase (G1, S, G2 and M) for 72 hours in SKLMS1 cells with and without ADI-PEG20 treatment corresponding to Figure 1H and I. Data are mean ± SD (*n* = 3).
