# Supplementary material for: BCL-XL Protects ASS1-Deficient Cancers from Arginine Starvation–Induced Apoptosis
Source: Clin Cancer Res. 2025 Feb 3;31(7):1333–45. doi: 10.1158/1078-0432.CCR-24-2548 (PMC11964295; doi:10.1158/1078-0432.CCR-24-2548)
Supplement: Supplementary Figure S2 — ADI-PEG20 and A1331852 combination treatment disrupts BCL-XL:BAX interaction in vitro [file ccr-24-2548_supplementary_figure_s2_suppfs2.pdf]

SUPPLEMENTARY FIGURE 2

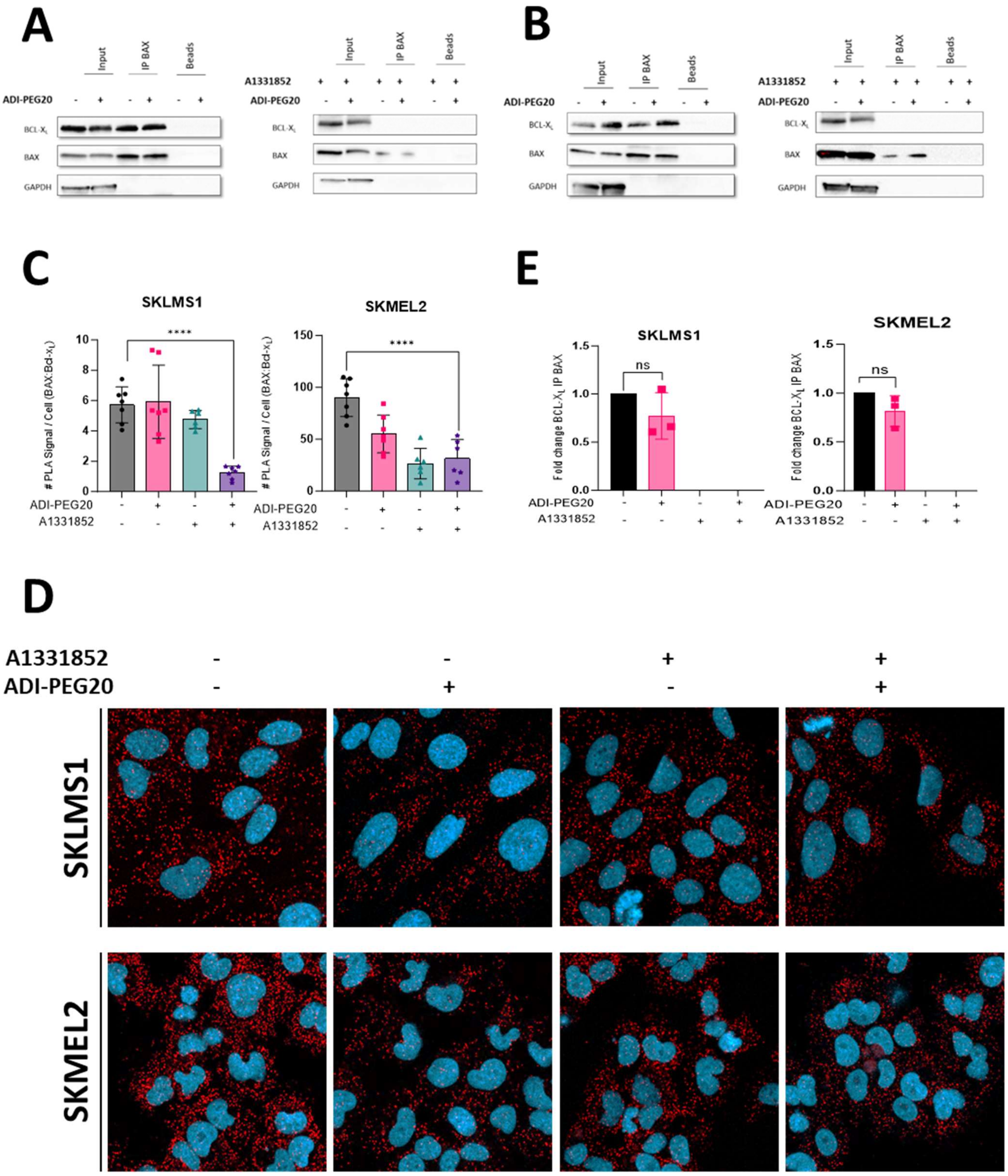

Supplementary Figure 2.

ADI-PEG20 and A1331852 combination treatment disrupts BCL-XL:BAX interaction *in vitro*. **A** (SKLMS1)**, B** (SKMEL2)**,** Immunoprecipitation (IP) analysis of BCL-XL and detection of co-immunoprecipitated/BCL-XL-bound BAX upon exposure to ADI-PEG20, A1331852, or combination treatment after 6 hours for SKMLS1 cells and after 12 hours for SKMEL2 cells, respectively. **C,** PLA signal quantified using Image J (Fiji). **D,** Images of SKMLS1 and SKMEL2 cells with or without ADI-PEG20, A1331852 or combination of both treatments. Each Proximity Ligation Assay (PLA) signal (red) is indicative of one detected BCL-XL: BAX interaction event. Nuclei (blue) are stained with Hoechst 33342. **E,** Band densitometry analysis of immunoprecipitated BAX in SKLMS1 and SKMEL2 cells. *,P< 0.05; **, *P* < 0.01; ***, *P* < 0.001; ****, *P* < 0.0001.
