# Supplementary material for: BCL-XL Protects ASS1-Deficient Cancers from Arginine Starvation–Induced Apoptosis
Source: Clin Cancer Res. 2025 Feb 3;31(7):1333–45. doi: 10.1158/1078-0432.CCR-24-2548 (PMC11964295; doi:10.1158/1078-0432.CCR-24-2548)
Supplement: Supplementary Figure S3 — BAX knockdown represses cell death induced by the combined treatment of ADI-PEG20 and A1331852 [file ccr-24-2548_supplementary_figure_s3_suppfs3.pdf]

SUPPLEMENTARY FIGURE 3

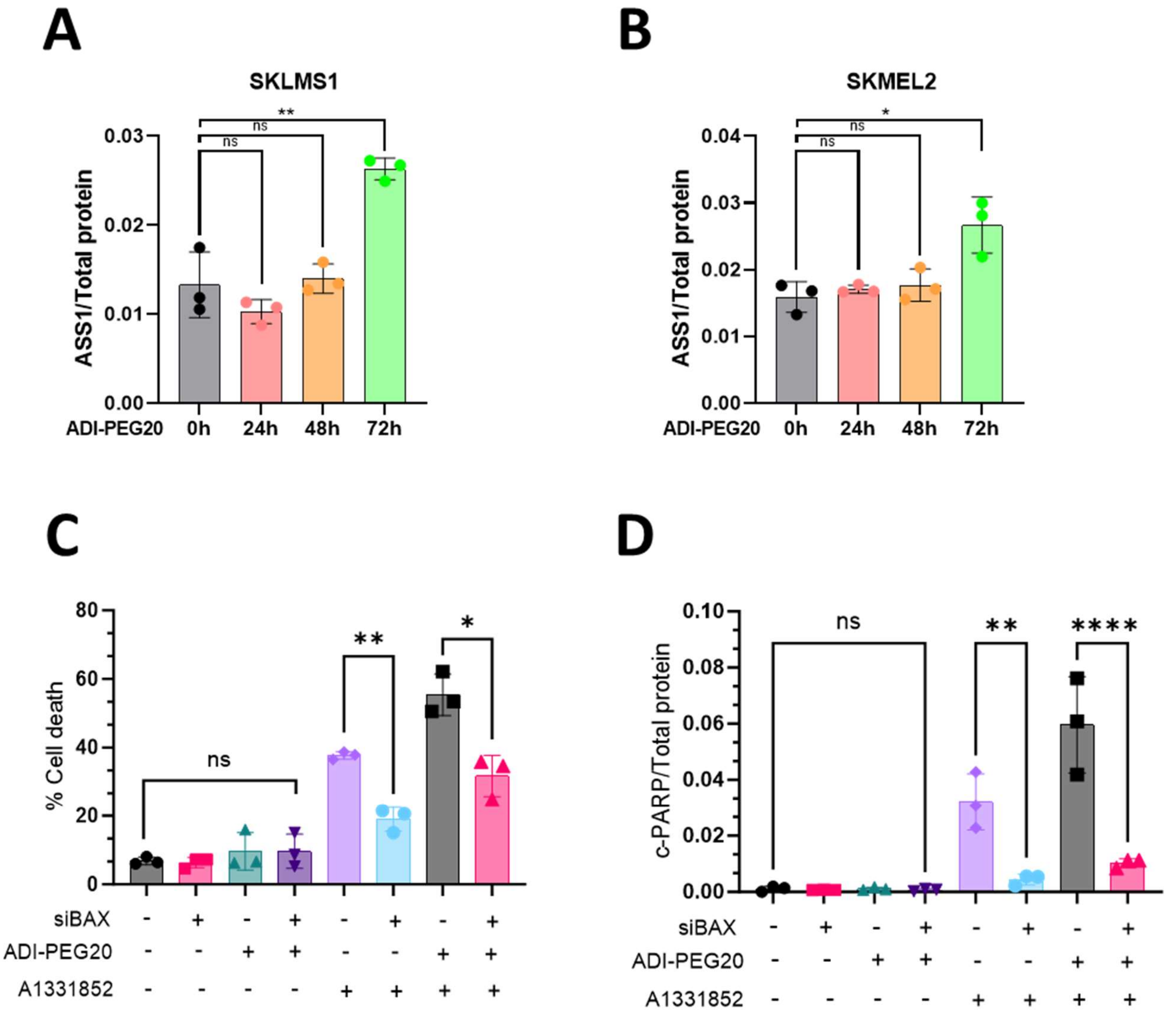

Supplementary Figure 3.

BAX knockdown represses cell death induced by the combined treatment of ADI-PEG20 and A1331852. **A, B,** ASS1 expression after 0, 24, 48, 72 hours of ADI-PEG20 treatment. **C,** Percent cell death in SKLMS1 cells after 24 hours of ADI-PEG20, A1331852, or combination of both treatments after *in vitro* silencing of *BAX*. **D,** c-PARP expression in SKLMS1 cells after 24 hours of ADI-PEG20, A1331852, or combination of both treatments after *in vitro* silencing of *BAX*. Two-tailed paired *t* tests for **A**-**D**. *,P< 0.05; **, *P* < 0.01; ***, *P* < 0.001; ****, *P* < 0.0001.
