# Supplementary material for: BCL-XL Protects ASS1-Deficient Cancers from Arginine Starvation–Induced Apoptosis
Source: Clin Cancer Res. 2025 Feb 3;31(7):1333–45. doi: 10.1158/1078-0432.CCR-24-2548 (PMC11964295; doi:10.1158/1078-0432.CCR-24-2548)
Supplement: Supplementary Figure S4 — Arginine starvation leads to MCL1 repression through CDK2 repression [file ccr-24-2548_supplementary_figure_s4_suppfs4.pdf]

SUPPLEMENTARY FIGURE 4

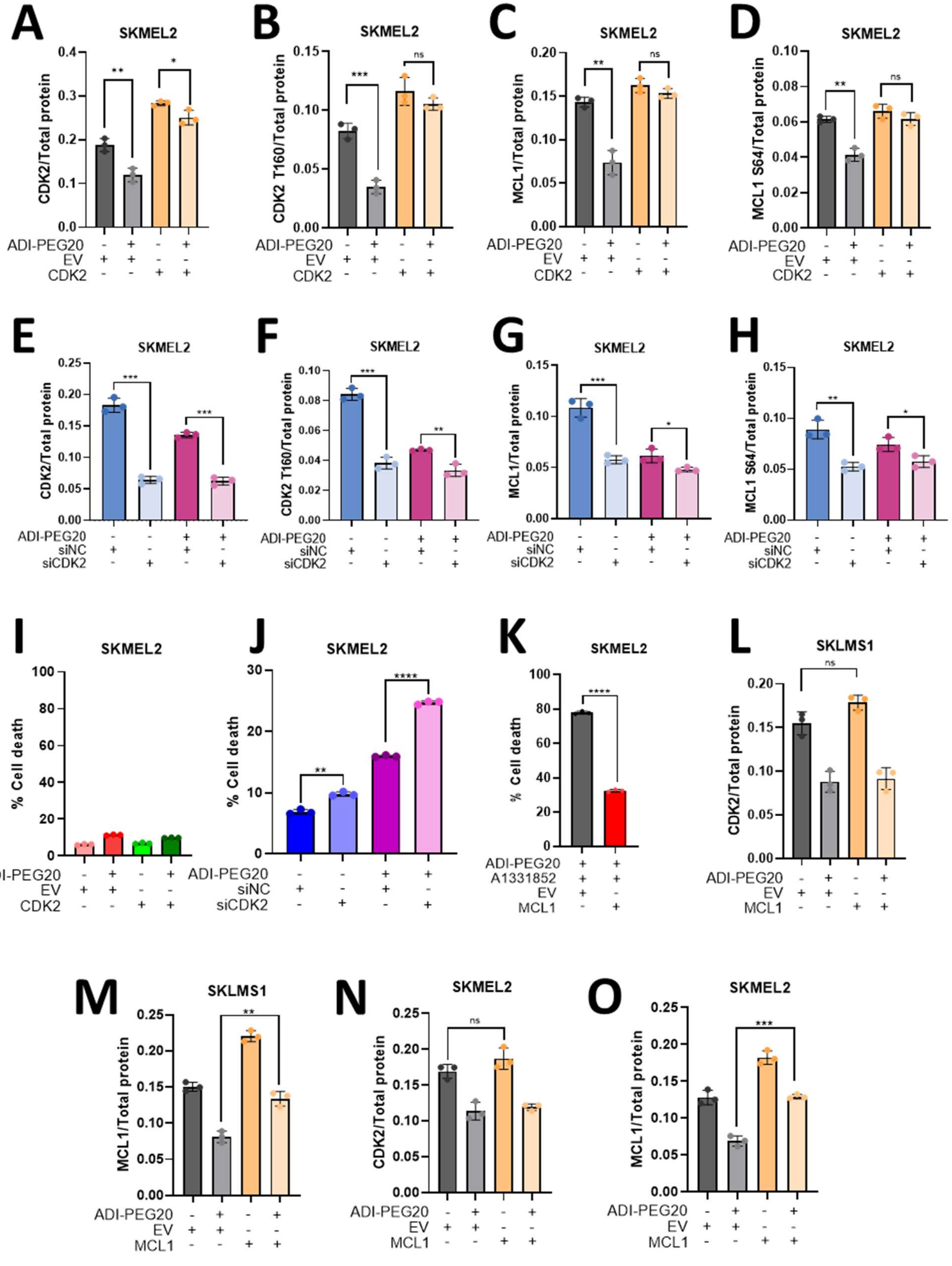

Supplementary Figure 4.

Arginine starvation leads to MCL1 repression through CDK2 repression. **A-D,** CDK2 protein expression (A), CDK2 T160 phosphorylation (B), MCL1 protein expression (C), and MCL1 S64 phosphorylation (D) in SKMEL2 cells overexpressed CDK2 or empty vector (EV) and subsequently treated with ADI-PEG20. **E-H,** CDK2 protein expression (E), CDK2 T160 phosphorylation (F), MCL1 protein expression (G), and phosphorylation of MCL1 S64 (H) in SKMEL2 cells knock downed CDK2 or non-targeting control (NC) and subsequently treated with ADI-PEG20. **I,** percent of cell death of CDK2 overexpressed SKMEL2 cells with ADI-PEG20 treatment at 24 hours. .**J,** percent of cell death of CDK2 knockdown SKMEL2 cells with ADI-PEG20 treatment at 24 hours. **K,** percent of cell death of MCL1 overexpressed SKMEL2 cells with combined treatment of ADI-PEG20 and A1331852 at 24 hours. **L, M,** CDK2 (L) and MCL1 (M) protein expression in SKLMS1 cells overexpressed MCL1 or empty vector and subsequently treated with ADI-PEG20. **N, O,** CDK2 (N) and MCL1 (O) protein expression in SKMEL2 cells overexpressed MCL1 or empty vector and subsequently treated with ADI-PEG20. Two-tailed paired *t* tests for **A**-**O**. *,P< 0.05; **, $P < 0.01$; ***, $P < 0.001$; ****, $P < 0.0001$.
