# Supplementary material for: BCL-XL Protects ASS1-Deficient Cancers from Arginine Starvation–Induced Apoptosis
Source: Clin Cancer Res. 2025 Feb 3;31(7):1333–45. doi: 10.1158/1078-0432.CCR-24-2548 (PMC11964295; doi:10.1158/1078-0432.CCR-24-2548)
Supplement: Supplementary Figure S5 — ADI-PEG20 and A1331852 synergically induce cell death. [file ccr-24-2548_supplementary_figure_s5_suppfs5.pdf]

SUPPLEMENTARY FIGURE 5

A **SKLMS1 24h**

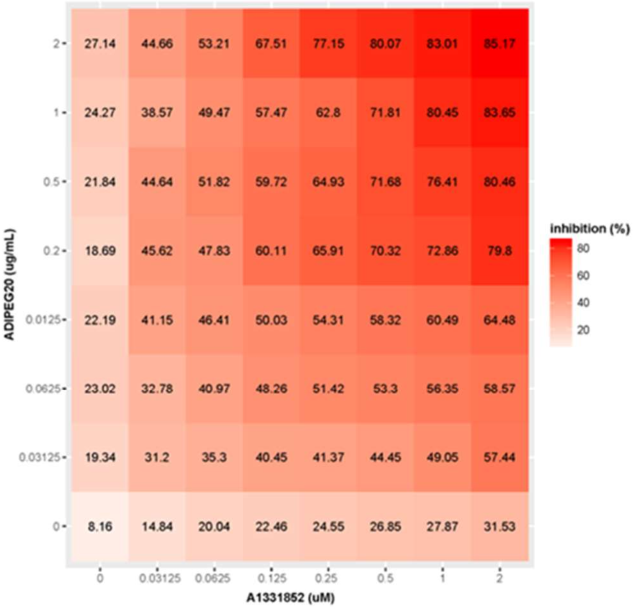

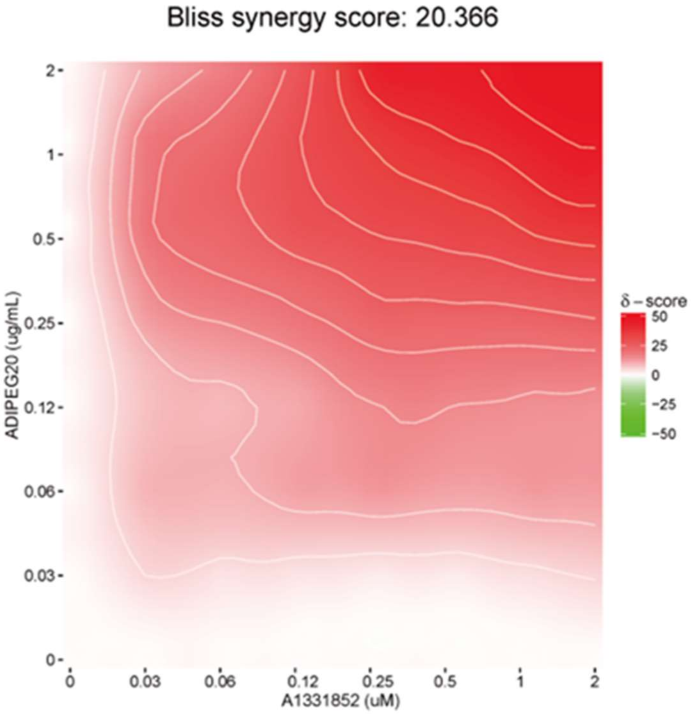

B **SKMEL2 24h**

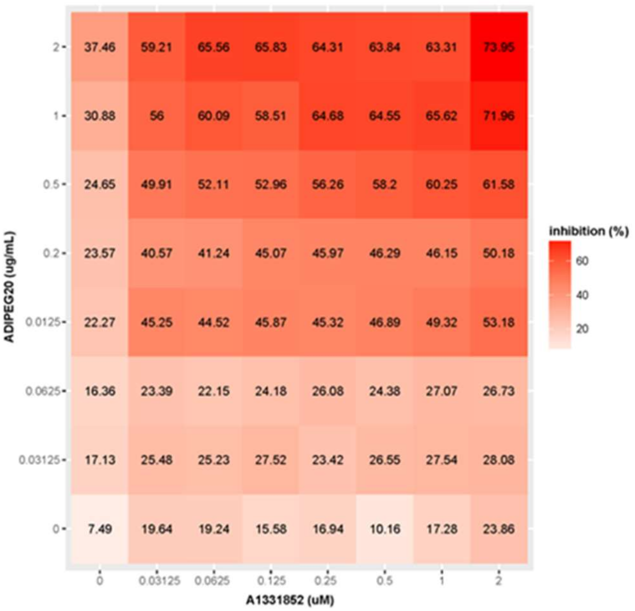

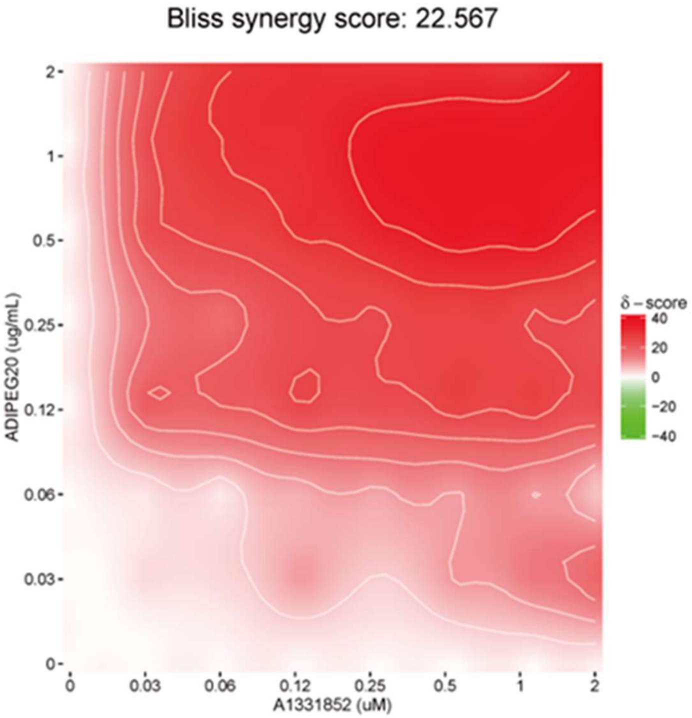

Supplementary Figure 5.

ADI-PEG20 and A1331852 synergically induce cell death. **A, B,** Analysis of the synergy between ADI-PEG20 and A1331852 at 24 hours in SKLMS1 and SKMEL2 cells respectively.
